# The roles of cognitive dissonance and normative reasoning in attributions of minds to robots

**DOI:** 10.1186/s41235-024-00604-3

**Published:** 2024-12-12

**Authors:** Lewis J. Baker, Hongyue Li, Hugo Hammond, Christopher B. Jaeger, Anne Havard, Jonathan D. Lane, Caroline E. Harriott, Daniel T. Levin

**Affiliations:** Vanderbilt University, Nashville, USA

## Abstract

As a wide variety of intelligent technologies become part of everyday life, researchers have explored how people conceptualize agents that in some ways act and think like living things but are clearly machines. Much of this work draws upon the idea that people readily default to generalizing human-like properties to such agents, and only pare back on these generalizations with added thought. However, recent findings have also documented that people are sometimes initially hesitant to attribute minds to a machine but are more willing to do so with additional thought. In the current experiments, we hypothesized that these attribution-increasing reconsiderations could be spurred by situation-induced cognitive dissonance. In two experiments, participants completed a belief activation exercise designed to induce cognitive dissonance (writing arguments for or against prominent beliefs), viewed a video of an ambiguously intentional robot, and completed measures of cognitive dissonance. In both experiments, cognitive dissonance was associated with increased attributions of mind to the robot. Our findings provide evidence that people sometimes increase their attributions of minds when experiencing cognitive conflict, but also that activation of change-inducing concepts may impact attributions of a mind without producing conscious cognitive conflict in participants.

**Significance Statement**

People’s understanding of technological agents such as robots is important not only because it may help them effectively use these agents, but also because these understandings may affect people’s willingness to accept these agents for different roles in society. One key inference that people are challenged to make about these agents is the degree to which they do, or do not, think like people. In some circumstances, people may initially infer that these agents are very much like people but when they get additional experience with the agent, come to realize that they are different. But in other circumstances, people may initially believe that artificial agents are very different from people and then revise their understanding by attributing more mind to an agent as their initial ideas are challenged. In this paper, we document the latter pattern of thinking and find that experiences of cognitive conflict can predict increased attributions of a mind to a robot. These findings indicate that changes to people’s understanding of robots may be predicted by the amount of cognitive conflict they experience, and this may inform interventions that address or induce cognitive conflict in the service of helping people learn more about a wide range of technological agents.

## Introduction

Over the past quarter century, a steady stream of intelligent technologies has rapidly transformed our society, affecting everything from our living room floors to our highways. According to projections, the 2021 global market for robotic vacuum cleaners was $11.97 billion dollars, and the 2028 market will be worth $50 billion USA dollars (Fortune Business Insights, 2022). In 2023, the autonomous car market is valued at approximately $33 billion USD (Mordor Intelligence, 2023). Generative artificial intelligence (GenAI) such as ChatGPT (OpenAI, 2023), which has the capability to create text, images, code, and other media from user prompts, is also becoming rapidly adopted in several sectors including education, research, finance, and information technology (Baidoo-Anu & Owusu Ansah, 2023; Goldfarb et al., 2020). Interaction with these technologies is inevitably informed by people’s understanding of how they operate, and researchers have argued that this understanding is strongly influenced by a default tendency to anthropomorphize technological agents that is lessened only with additional thought (Epley et al., 2007). However, as we will review below, there is good reason to think that people do not always default to anthropomorphize. Sometimes people by default strongly distinguish between the goal-directed actions of living intentional agents and the more rote actions of nonliving mechanical agents (Levin et al., 2013a, 2013b), and with further consideration, this difference may lessen (Levin et al., 2012). In the current paper, we explore two factors that might influence the degree to which the thoughts that accompany simple observations of robotic agents increase or decrease attributions of mind: cognitive dissonance and activation of concepts about counter-intuitive agents. In two experiments, we uncover evidence consistent with the hypothesis that self-reported cognitive dissonance is associated with increased attribution of minds. We also find evidence that activation of counter-normative concepts about a supernatural agent such as God increases attributions of mind, but only for a subset of attributions.

Before we begin, it is important to situate these results with respect to concepts of minds, agency, and anthropomorphism. In these experiments, we assess the mental capabilities that participants attribute to a robot broadly, and so we define mind attribution as the judgment that an agent “possesses mental states such as beliefs, desires, and complex emotions” (Morewedge et al. 2013 p. 1195), and we also include the attribution of mental processes such as memory and planning (Gray, Gray, and Wegner, 2007). A key question concerns the relationship between mind attribution and anthropomorphism. Epley et al (2007) state that “Imbuing the imagined or real behavior of non-human agents with human-like characteristics, motivations, intentions, and emotions is the essence of anthropomorphism.” Conceivably, mind attribution might in some ways be nonanthropomorphic if people can imagine non-human mental processes that nonetheless can be considered to embody mental processes such as beliefs, emotions, and planning. However, this is probably rare, and most researchers, including Morewedge et al. (2013) and Gray et al. (2007), argue that strong attributions of mind are closely associated with humanness (see also Guzman, 2020; Koban and Banks, 2024). Also, as reviewed below, Barrett and Landman (2007) propose that detection of agency (that is, relatively immediate detection of goal-directedness) activates inferences driven by theory of mind which constitutes a set of skills that readily support inferences about human-like, or at least intentional, mental states. Thus, the discussion below, and our measures, assumes that strong mind attribution is an important component of anthropomorphism, and that agency is defined by either the detection of goal-directedness or inferences about mental processes that most directly support goal-directed, or self-initiated action. As such agency inferences would be considered a subset of mind attributions (e.g., Gray, Gray, and Wegner, 2007).

## Anthropomorphize first, ask questions later

In a variety of contexts, people attribute minds characterized by active cognitive processes, goals, and intentions to robots and other technological entities (e.g.,Jipson & Gelman, 2007; Kahn et al., 2004, 2012; Melson et al., 2009; Nass & Moon, 2000). These findings are part of a larger literature exploring anthropomorphic attributions to animals (Carey, 1985), animated shapes (Abell et al., 2000; Heider & Simmel, 1944), gods (Barrett & Lanman, 2008; Heiphetz et al., 2016), and more (for review, see Epley et al., 2007). By attributing goals and intentions to a non-human agent (adopting an *intentional stance*), people assume it follows certain logical behaviors, and is rational, predictable, and consistent (Dennett, 1989).

Generally, anthropomorphizing can be very useful. It enables us to make sense of unfamiliar processes and behavior (Waytz et al., 2010), helps satisfy social and emotional needs (Barrett & Lanman, 2008; Epley et al., 2008; Guthrie, 1995), may increase trust in artificial agents (Waytz et al., 2014), increases acceptance toward moral decisions made by agents (Gray et al., 2012), provides cognitive benefits such as increased selective attention (Spatola & Huguet, 2021; Spatola et al., 2020), improved memory for events (Baker et al., 2018), and may shape learning outcomes (Hymel et al., 2011; Jaeger et al., 2019). Given these benefits, several researchers have proposed that people anthropomorphize entities that exhibit even minimal cues of agency such as movement without a clear physically caused origin (Barrett & Lanman, 2008; Epley et al., 2007).

A variety of findings demonstrate how readily people attribute minds to apparently intelligent machines. For example, participants who engage in minimal interactions with computers apply a variety of social norms to them (Nass & Moon, 2000), participants (both children and adults) who interact with robots are often very willing to attribute minds to them (Jipson & Gelman, 2007; Kahn, 2006; Kahn et al., 2012; Melson et al., 2009), and even infants seem to attribute goals to ambiguous agents based on minimal behavioral cues (Johnson, 2003). In a similar vein, Barrett and Lanman (2008) propose that attributions of minds rely on a “hyperactive agency detection device,” which can explain why people attribute agency to the causes of ambiguous events, and more generally can explain why people are predisposed to believe in supernatural agents such as God. Importantly, this attribution is assumed to be based both on a low-threshold identification of agents as well as a highly likely “immediate” default to activate Theory of Mind-based inferences about beliefs and goals.

However, as many of these models emphasize, these attributions are only defaults and can be modified with additional motivated thought. Epley et al. (2007) propose that several factors can induce people to rethink their initial judgments, either paring it back or resisting correction. First, “elicited agent knowledge” reflects the availability of knowledge about nonanthropomorphic forms of thought. Second, “effectance motivation” reflects how a situation may demand successful interaction with an agent that cannot rely on default judgments. Finally, variations in “sociality motivation” may prevent lessening of mind attributions when individuals who are lonely desire social contact. Similarly, Barrett and Lanman (2008) propose that reflective inferences can occur with sufficient motivation, and that these can modify initial non-reflective defaults.

## Selective mind attribution: distinguish, then attribute agency only when warranted

Existing models of mind attribution do a good job of explaining an array of important findings. However, there are several reasons to believe that they may not capture the full range of judgments about agents. First, a large amount of developmental research demonstrates that in infancy children distinguish living things from nonliving things (Opfer & Gelman, 2010), and that this basic categorical difference is elaborated into differentiated expectations about the goal-directed behavior of living things vs the more rote behavior of nonliving things (Opfer & Siegler, 2004). This early distinction is elaborated in the first few years of life into an understanding of the desire- and belief-driven behavior of intentional agents (Wellman, 2014; Woodward, 2005). Although it is plausible that in many situations the attribution of beliefs and goals may be a default, even preschoolers can learn about novel cues such as remote controls that signal non-agency in moving objects such as robots (Somanader et al., 2011). Thus, it would be surprising if adults lacked a readily available and deeply embedded framework to apply in understanding the actions of nonliving things. Consistent with this possibility, in several studies, participants seem initially reluctant to anthropomorphize machines and become more willing to do so with deeper consideration (Hymel et al., 2011; Levin et al., 2008, 2012, p. 201; Levin et al., 2013a, 2013b; Levin et al., 2013a, 2013b). We refer to this as a “selective attribution” pattern, as participants sometimes seem to default to a strong contrast between intentional and nonintentional agents that may loosen with additional thought.

Levin et al., (2013a, 2013b) documented selective attribution in a series of experiments that asked participants to predict how a human, a desktop computer, and a robot (which was presented in an anthropomorphic form) would behave in different scenarios. For example, in one scenario, participants were told an agent had selected one of two objects sitting on a tabletop. Then, the locations of the objects were switched, and participants were asked which object the agent would select: the same one that was now in the new location, or the previously unselected object that now sat in the location of the previously-selected object. If participants attribute human-like goals to these agents, they might predict that the agent will select the same object in the new location, but if they do not attribute goals, they might predict that the agent will either select the new object at the old location or make predictions at chance (as do infants in studies of their goal-directed inferences about people; Woodward, 2009). Participants strongly distinguished humans from both robots and computers, and simple cues such as human form or simple human-like reaching actions did not increase the likelihood of attributing human-like goal-directedness to robots. Participants attributed more goal-directedness to the robot only after they were asked to focus closely on a robot making a series of goal-directed choices between two objects. Using similar behavioral prediction scenarios, Levin et al. (2012) found that participants who responded more quickly drew sharper distinctions between humans and machines than participants who responded more slowly. In contrast with the generalize-then-pare back pattern of findings, these studies suggest that, in some contexts, deeper consideration leads people to anthropomorphize technology more.

## The impacts of cognitive dissonance and activation of explicit thoughts about supernatural agents on attributions of agency.

Our aim in this paper is to detail how some key cognitive processes may induce mind attribution reconsiderations. First, we hypothesize these reconsiderations are spurred by cognitive dissonance. Although cognitive dissonance has its roots in the attitude change literature (for review, see Elliot & Devine, 1994; Festinger, 1962), we use the term in a slightly different sense in this context. Specifically, we define cognitive dissonance as awareness of conflicting concepts, or with concepts that conflict with experiences, often associated with discomfort. Thus, cognitive dissonance often signals a disconnect between our knowledge and apparent reality, indicating a need to know more (see, e.g., Jonassen & Land, 2012). People tend to experience cognitive dissonance when things do not make sense for them and alleviate that dissonance via conceptual change (e.g., Hewson & Hewson, 1984). In the context of human–technology interaction, alleviating that dissonance may entail using familiar, anthropomorphic inferences—in the form of increased mind attributions—to help one “understand” the technology (e.g., adopting a schema that explains apparent behavior in anthropomorphic terms; Baker et al., 2018). Importantly, lessening dissonance may also involve paring back mind attributions (e.g., if the technology is humanoid in appearance but has limited capabilities or functions very poorly during an interaction). Such dissonance may prompt a change in either one’s behavior or one’s conceptualization of the entities inducing the dissonance.

A similar concept in the learning science literature is “cognitive disequilibrium”—perceived inconsistencies in material that lead students to deepen their analysis of a problem (Graesser et al., 2005). This sort of cognitive dissonance or disequilibrium could be triggered when a person’s interaction with a technological entity is inconsistent with their experience or expectations—e.g., if the technology fulfills a role or function traditionally reserved for humans, if it behaves or responds in an unpredictable manner, or, more generally, if it performs surprisingly well or surprisingly poorly.

One previous experiment has explored the association between cognitive dissonance and attributions of minds to robots. Levin et al., (2013a, 2013b) developed a simple 6-item questionnaire assessing cognitive dissonance (participants responded to items such as “I was always certain about my responses,” and “If I were allowed to, I would go back and change some of my responses”), and had participants interact with a robot in a realistic hazardous materials scenario in which the participant worked with the robot, moving among simulated victims, reporting via radio to a remote operator about the medical condition of the people. In a comparison condition, subjects worked with another person instead of a robot. In the human–robot condition, but not the human–human condition, increased cognitive dissonance was associated with significantly less intentional attributions about computers. At first blush, the direction of the effect may seem to run counter to the selective agency hypothesis—*increased* thought and cognitive conflict accompanied *fewer* anthropomorphic attributions. As suggested above, this is plausible if the interaction reveals limits to the technology. Consistent with this possibility, the robots in this scenario were minimally intelligent and could not perceive anything about their surroundings. Participants working with the robot were required to report observable signs to a remote partner, making it clear that the robot was incapable of doing this. Thus, participants were likely disappointed by the lack of capability exhibited by the robots. This disappointment was reflected in post-experiment teammate evaluation ratings of the robot partner, which indicated that participants found the robot to be less trustworthy and less able to communicate than the human partner.

Accordingly, in the present experiments, we tested for associations between cognitive dissonance and mind attribution in conditions where the robot’s actions would not so starkly reveal its limits. We propose that cognitive dissonance may, in some circumstances, induce increased mind attribution. We tested our hypothesis in two online experiments assessing participants’ attribution to a robot they viewed in a video. To increase the likelihood of dissonance-inducing cognitions, we added a second key cognitive process to our experimental context. We asked participants to engage in nominally normative and non-normative reasoning about a distinctive agent: God. Previous studies imply that adult participants are more likely to attribute agency to a novel agent when primed with concepts about atypical agents. In particular, asking participants to consider divine or supernatural agents has been shown to expand attribution of causal forces to relatively coincidental actions (Nieuwboer et al., 2015). However, it is also possible that this form of reasoning may induce more selective attributions of mindedness, especially if participants are induced to make arguments counter to the prevailing norm of belief in God. As a starting point, we therefore hypothesize that activation of non-normative religious beliefs will increase dissonance and in turn increase attributions of agency. In these experiments, we attempted to activate these beliefs by asking some participants to give reasons one might *not* believe in God, and compared these participants with another group who were asked to give reasons that one might believe in God. Not all of our participants will necessarily find giving reasons not to believe in God equally conflict-inducing, especially if they are atheists. However, we recruited participants from the USA, where belief in a God is normative—83% of adults are either “absolutely certain” or “fairly certain” that God exists (Pew Research Center, 2023), so our initial analysis assumed that arguing against the existence of God would induce increased dissonance both because it may conflict with personal beliefs and because it is counter-normative, so this initial analysis used condition is a main effect predictor. However, we added a measure of self-report religiosity as a covariate both to use as a main effect predictor in the initial model, and to use as an interaction predictor in a follow-up to test whether arguing against one’s specific beliefs, as opposed to arguing for globally counter-normative beliefs, would induce dissonance.

## Experiment 1

Experiment 1 investigated the basic association between cognitive dissonance and mind attributions about a robot. Here, participants viewed a video of a robot engaging in an ambiguous interaction with a person and then completed questionnaires about their mind attributions regarding the robot and feelings of cognitive dissonance. To activate these different reasoning processes, participants completed a brief belief activation exercise (writing 5–8 sentences giving reasons to believe or not believe in God), before viewing the robot video. The hypothesis is that cognitive dissonance will lead to increased attributions of agency, and that cognitive dissonance will be increased as participants struggle to make meaning from the prompt, especially when they need to argue counter-normatively. To test our hypotheses, we conducted a simple path analysis. The analysis included exogenous variables representing the impact of condition (arguing for or against the existence of God), self-reported religiosity, and the interaction between these two variables. We tested whether cognitive dissonance would be impacted by these variables and would in turn predict the attribution of agency to an example robot.

## Methods

### Participants

Participants (*N* = 123, mean age = 33.8 years, *SD* = 11.4; 60 male, 62 female, 1 unreported gender) were recruited via Amazon’s Mechanical Turk website for a $0.50 compensation. One participant was removed for not completing the entire experiment, leaving 61 participants in each condition. Mechanical Turk has been validated for use in cognitive science research (Germine et al., 2012; Paolacci et al., 2010). Although there may be some concern that this platform can include misrepresented and/or nonsensical data (for review, see Fowler et al., 2022), recent reviewers validating the platform have replicated earlier ones (De Lurgio et al., 2023), and we also note that the vast majority of participants (119 of 122, as agreed by independent raters) effectively described the events in the video, strongly suggesting they took the time to view it. Ethical approval was obtained from Vanderbilt University’s Institutional Review Board.

### Procedure

Upon giving digital consent, participants proceeded to one of two narrative prompt conditions. Participants were instructed that we were interested in “how people take sides on controversial issues” and were told “while we know people have a variety of beliefs, please defend the statement below to the best of your ability.” They were then asked to write 5–8 sentences to one of two randomly assigned prompts: “Why might someone believe in God?” or “Why might someone **not** believe in God?”

Upon completion of the prompt, participants viewed a 34 s video (available online at https://vimeo.com/889162682) of an actress appearing to interact with a robot. In the video, a woman found a door locked, looked for her keys, heard a noise off-screen, and turned to find a small robot holding a key ring. The robot moved off-screen and she followed.

Participants wrote 4–8 sentences describing what they saw in the video. They then completed a 5-item mind attribution questionnaire, where they ranked on a seven-point Likert scale the degree to which the robot “has intentions,” “does not have free will” (reverse scored), “has consciousness,” “does not experience emotions” (reverse scored), and “has a mind of its own.” Then, participants completed a demographic survey, including questions about religiosity. This survey asked participants to rate their agreement with four statements on a seven-point Likert scale, anchored between “completely disagree” and “completely agree”: “I believe in God,” “My religious faith is very important to me,” “My faith provides guidance in my day-to-day living,” and “I feel confident in discussing the history and major teachings of my faith.” Two additional questions asking: “How often do you pray?” and “In the last 12 months, how often have you attended religious services, not including weddings, baptisms, or funerals?” were also included and rated on a six-point scale, ranging from “Never” to “Daily.” These items were transformed to a seven-point scale, and religiosity was derived as the average of all six items.

Participants concluded with a cognitive dissonance survey adopted from Levin et al., (2013a, 2013b). This survey included 6 items, rated on a seven-point Likert scale, anchored between “completely disagree” and “completely agree.” The items were: “Sometimes I was uncomfortable answering these questions,” “At times I worried that some of my answers were inconsistent with my other answers,” “If I were allowed to, I would go back and change some of my responses,” “Some of the answers I gave in this experiment were inconsistent with my previous beliefs about the subject,” “I was always certain about my responses” (reverse scored), and “I never had difficulty putting together all of the facts in this experiment.”

## Results

Cognitive dissonance and attributions of agency were both scored on a 7-point Likert scale. The mean cognitive dissonance score was 2.02 (*SD* = 0.95; range of 1 – 4.83, Cronbach’s α = 0.65). A confirmatory factor analysis on the cognitive dissonance items testing a single factor model demonstrated mediocre fit (*CFI* = 0.821; *RMSEA* = 0.149). A correlation matrix of the items demonstrated that almost all intercorrelations were in the correct direction and significant. One issue with the scale is that scores in this administration were overall low, so many of the individual items were right-skewed. We present results based on the full scale here, then present additional analysis on both administrations of the scale after the main analysis of Experiment 2. To preview, though, none of the substantive conclusions changed when substituting individual items into the analysis.

The mean mind attribution score was 2.45 (*SD* = 1.39; range: 1 – 7, Cronbach’s α = 0.84). These questions had not been previously tested together so we assessed them using an exploratory factor analysis, and this yielded a single factor (eigenvalue = 3.104, 53% of variance explained). The mean religiosity score across participants was 3.02 (*SD* = 1.97, range: 1 – 7, Cronbach’s α = 0.90). These questions also yielded a single factor (eigenvalue = 4.566, 72% of variance explained). Roughly half (45.9%) of participants identified as religious, 32.0% identified as spiritual but non-religious, and 22.1% as neither religious nor spiritual. To assess the effects of our belief activation task and cognitive dissonance on attributions of agency, we tested a simple mediation model using JASP version 0.18.1 (2023). Included in this model were condition (dummy coded as pro belief prompt = 1, con belief prompt = 2) and religiosity, and a condition x religiosity interaction as predictors, cognitive dissonance as a mediating variable, and attributions of agency as our outcome variable. Effect sizes for this model were calculated using bootstrapped confidence intervals with 1000 iterations.

**Fig. 1:**
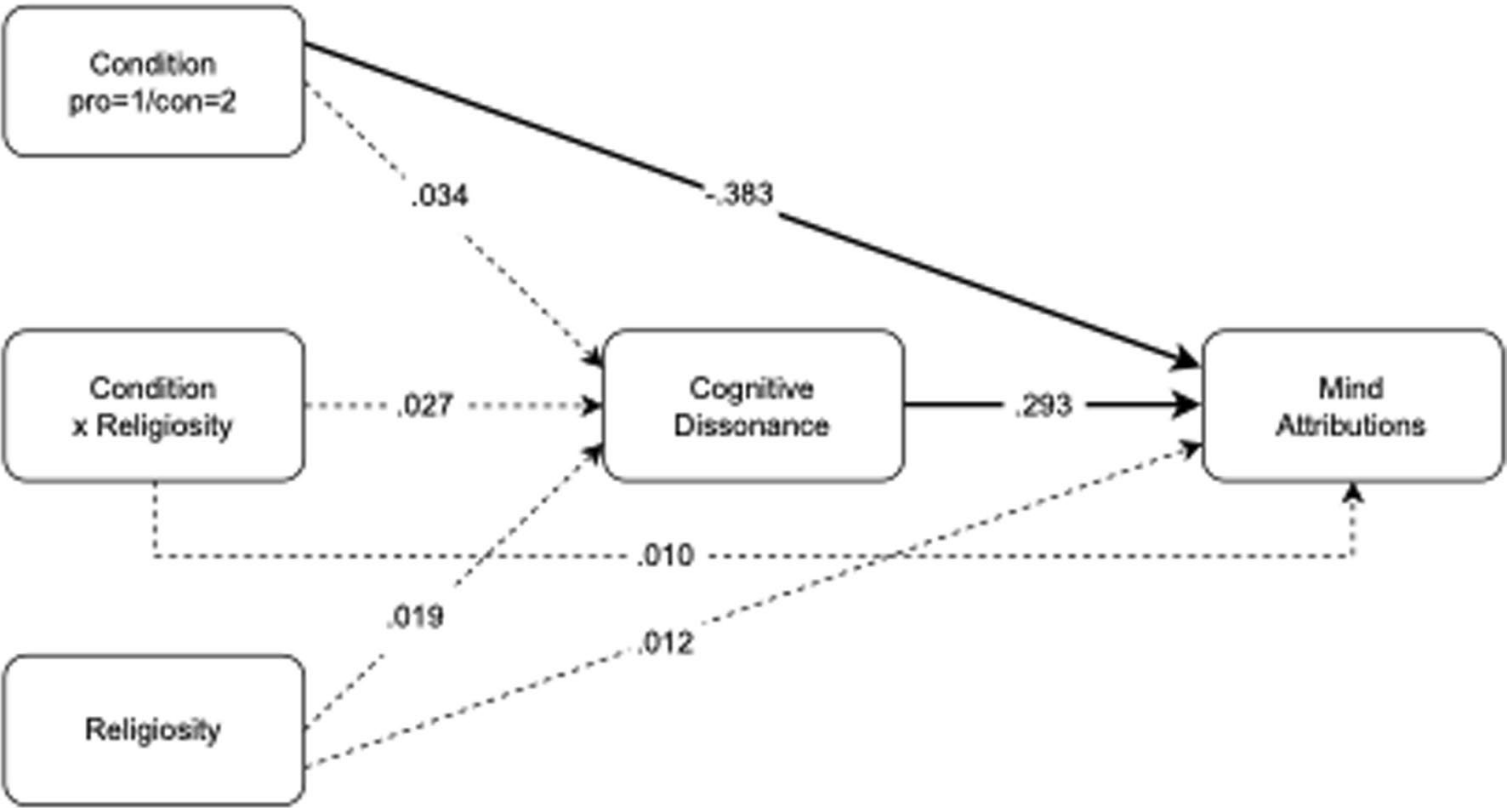
Mediation model for Experiment 1. Bold lines display significant effects and dashed lines represent non-significant effects. Model coefficients are standardized beta estimates. Condition is dummy coded as pro belief prompt = 1, con belief prompt = 2

As shown in Fig. 1, there was a significant direct effect of condition on mind attributions (Standardized Beta = -0.383; 95% *CI*[-0.719, -0.047], *z* = -2.234, *p* = 0.026): arguing against reasons to believe in God was associated with lower attributions of agency. Further, as anticipated, cognitive dissonance was a significant positive predictor of attributions of agency (Beta = 0.293; 95% *CI*[0.127,0.459]; *z* = 3.457; *p* < 0.001). Cognitive dissonance was not significantly impacted by condition (Beta = 0.034; 95% *CI*[-0.325,0.393]; *z* = 0.182, *p* = 0.856) religiosity (Beta = 0.019; 95% *CI*[− 0.073,0.111]; *z* = 0.381, *p* = 0.703), or the condition x religiosity interaction (Beta = 0.027; 95% *CI*[− 0.157,0.210]; *z* = 0.288, *p* = 0.774). The mean level of mind attributions by condition was 2.718 for participants who provided reasons to believe in God and 2.184 for those who provided reasons not to believe, *t*(120) = 2.158, *p* = 0.033, *d* = 0.391.

Overall, none of the mediated effects were significant (*p*’s > 0.68), and the only significant total effect was the one linking Condition to Mind Attributions (Beta = -0.373, 95% *CI*[-0.725,-0.021], *z* = -2.075, *p* = 0.038).

Some proponents of path modeling argue in favor of pruning non-significant effects from models, for simplicity and optimization, and recomputing model estimates (Zhu & Gupta, 2017), but we have not done this because it risks underemphasis on non-significant findings. However, we note that upon pruning non-significant effects, all currently significant estimates remain so.

## Discussion

Experiment 1 indicated that cognitive dissonance accompanied increased attributions of mind to a robot. This impact appears to be distinct from activating existing concepts about minds, given there was no direct effect of our agency belief activation prompt on dissonance ratings. Further, Experiment 1 did not find any direct impact of the belief prompt or religiosity on cognitive dissonance. As such, Experiment 2 will test whether attributions of mind are affected by dissonance around the prompt itself, or more general preexisting cognitive conflict. In addition, participants who generated reasons against believing in God attributed *less* mindedness to the robot than participants who generated reasons to believe in God. However, it is unclear whether this results from a tendency to argue against religious beliefs, or whether this may be a product of arguing against normative ideas more generally. This will be explored further in Experiment 2 through the addition of a non-religious control prompt.

## Experiment 2

Experiment 1 indicated that cognitive dissonance can increase attributions of mind, and that other forms of knowledge activation can also affect these attributions. However, counter to our predictions, dissonance did not mediate a relationship between belief activation and attributions of mind, because condition did not impact levels of dissonance. We replicated these findings in Experiment 2 and also tested a potential alternative explanation for the effect of dissonance on mind attributions. Although our measure of dissonance is designed to gauge cognitive conflict in response to the immediate context (recall that all of the items in the questionnaire refer to the current situation), it is possible that these responses are influenced by a broader tendency to experience cognitive conflict or uncertainty. For example, it is possible that some individuals may enter the experiment with a higher baseline level of uncertainty, and this general sense of conflict influences attributions of mind rather than conflict specifically induced by the current situation. To test this possibility, we asked participants in Experiment 2 to complete measures of cognitive dissonance at three points. Immediately following consent, participants completed a modified version of the cognitive dissonance questionnaire that referred to the experience of general cognitive conflict over the past “days and weeks.” Then, after completing the belief activation exercise, participants completed a second measure of dissonance relating to their experiences responding to beliefs about God prompt. Participants then viewed the robot video and completed the attribution of mind scale. They then responded to a third and final cognitive dissonance measure regarding the robot and their attributions to the robot.

This three-test approach to assessing dissonance has the added advantage of testing whether the link between dissonance and mind attributions was causally reversed. This might be true if making increased attributions of mind to a robot actually caused the dissonance we observed; this is possible because in Experiment 1 participants completed the dissonance measure after the mind attributions. However, if dissonance in response to the prompt predicts attributions before they are actually made, it would be unlikely the attributions themselves caused the dissonance we observed.

Additional goals of experiment 2 were to test whether religiosity and activated beliefs about God would again predict cognitive dissonance and attributions of mind, and to test the specificity and boundary conditions of associations between the prompt, cognitive dissonance, and mental state attributions. Conceivably, the findings in experimenter 1 rapply only to reasoning about an enigmatic agent (i.e., god) or reasoning about a highly personal and emotional topic (i.e., god’s existence). Therefore, in experiment 2 we asked some participants to make arguments about believing in God, and we asked other participants to list reasons that baseball should (or should not) be considered “America’s national sport.” We anticipated that the topic of baseball would be approached quite differently than the topic of god’s existence, with the topic of baseball presumably being less emotional and personal to most participants.

We also broadened our measure of mind attribution because the average level of mind attribution observed in Experiment 1 was relatively low. We therefore added items that more concretely referenced *basic* cognitive skills such as decision making, learning, and recognizing objects to possibly lower what might otherwise be a high threshold for attributions of exclusively higher-order, human-like mental properties, such as consciousness.

## Methods

### Participants

Participants (*N* = 115, *mean age* = 34.4 years, *SD* = 10.4, 62 male, 53 female) were recruited via Amazon’s Mechanical Turk website for a $0.50 compensation. Participants were distributed randomly across conditions, resulting in the following assignment: Pro God prompt, *n* = 29; Con God prompt, *n* = 30; Pro baseball prompt, *n* = 29; Con baseball prompt, *n* = 27.

### Procedure

Upon giving digital consent, participants completed an adapted measure of cognitive dissonance asking about their experience of cognitive conflict over the past “days and weeks.” As in Experiment 1, participants proceeded to write 5–8 sentences on one of four narrative prompts (this time including two prompts about baseball). The prompts were: “Why might someone believe in God,” “Why might someone **not** believe in God,” “Why might someone believe baseball is America’s national sport,” or “Why might someone **not** believe baseball is America’s national sport.” Upon completion of the prompt, participants again completed a measure of cognitive dissonance, this time referring to dissonance about the experience of the prompt. Finally, participants viewed the same video of an actress interacting with a robot as Experiment 1, described what they saw in the video, completed an expanded version of the attribution of agency questionnaire, and concluded with demographic questions and a final cognitive dissonance survey. This version of the agency questionnaire included a total of 21 items, each starting with “the robot…”: “has a mind of its own,” “has free will,” “has intentions,” “has consciousness,” “has emotions,” “makes decisions,” “uses strategies,” “considers alternatives before acting,” “knows things,” “can perceive his surroundings,” “gets frustrated,” “recognizes objects,” “understands language,” “has preferences,” “learns from mistakes,” “can solve new problems,” “can communicate” “can make plans,” “can navigate new spaces,” “has beliefs,” “understands abstract ideas.”

## Results

In Experiment 2, descriptive statistics for cognitive dissonance at each time point were: baseline (*M* = 4.12, *SD* = 1.21, range = 1 – 6.30, Cronbach’s α = 0.85), prompt (*M* = 2.96, *SD* = 0.92, range = 1.50 – 5.33, Cronbach’s α = 0.70), robot (*M* = 2.92, *SD* = 1.24, range = 1 – 5.83, Cronbach’s α = 0.87). Confirmatory factor analyses for each administration produced reasonable fits for one-factor solutions for some measures but not others. CFIT indices were reasonable for baseline dissonance (0.945), prompt dissonance (0.934), and robot dissonance (0.934). *RMSEA* fits were poor for baseline dissonance (0.222), the prompt dissonance (0.170), and robot dissonance (0.357). Descriptive statistics for mind attributions were *M* = 3.11, *SD* = 1.17, range = 1 – 6.20, Cronbach’s α = 0.95. An exploratory factor analysis produced two factors. The first (including “recognizes objects,” “can perceive his surroundings,” “uses strategies,” “can navigate new spaces,” “can solve new problems,” “makes decisions,” “can make plans,” “learns from mistakes,” “considers alternatives before acting,” “knows things,” “understands language,” “has intentions”) had an eigenvalue of 10.487, and it explained 30% of variance. The second factor (including “has emotions,” “has free will,” “has beliefs,” “gets frustrated,” “has consciousness,” “has a mind of its own,” “has preferences,” “understands abstract ideas”) had an eigenvalue of 2.765, and it explained 29% of variance. The item “can communicate” loaded on neither factor. The mean religiosity score across participants was 3.28 (*SD* = 1.92, range: 1 – 7, Cronbach’s α = 0.93). An exploratory factor analysis of the religiosity measure produced a one-factor solution (Eigenvalue = 4.419, 69% of variance explained). The same proportion of participants identified as religious (28.7%), and as spiritual but non-religious (28.7%), while 42.6% identified as neither religious nor spiritual.

To assess the effects of our variables on attributions of mind, we again tested a mediation model. Here, condition (Pro/Con belief prompt), topic (God/Baseball prompt), and religiosity were each included as predictors, with attributions of agency as the outcome variable again. In Experiment 2, cognitive dissonance was included separately as a mediator at the three time points: baseline, after the belief activation prompt, and after completing the agency attributions toward the robot. Because the interaction between religiosity and condition was non-significant in Experiment 1, we decided not to include the interaction in the final model of Experiment 2 when it again produced no significant paths. This was true both when the interaction was tested for all data (*p*’s > 0.07), in which case it was tested even when baseball was the topic, and only for the God condition (*p*’s < 0.11). This allowed us to focus the analysis and figures. Effect sizes were calculated using bootstrapped confidence intervals with 1000 iterations.

**Fig. 2:**
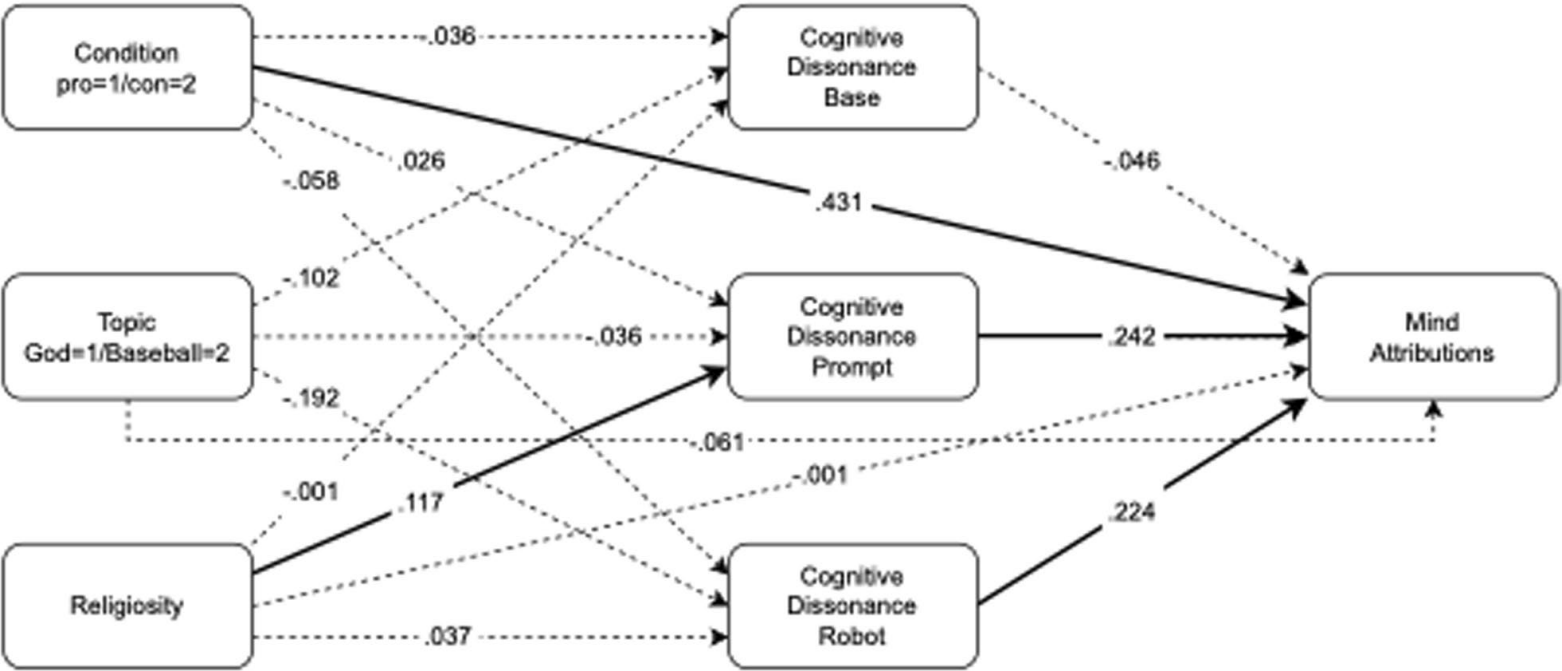
Mediation model for Experiment 2. Bold lines display significant effects and dashed lines represent non-significant effects. Model coefficients are standardized beta estimates

As shown in Fig. 2, the only direct effect from predictor to outcome variable was a link from condition on attributions of agency (Beta = 0.431, 95% *CI*[0.107, 0.755], *z* = − 2.606, *p* = 0.009). In contrast with Experiment 1, arguing against the prompt led to increased attributions of agency. To explore further whether increased attributions of agency are specific to negative arguments about beliefs in God (rather than simply adopting a negative stance), we conducted a 2 × 2 (topic x condition) ANOVA (analysis of variance) predicting attributions of agency. This revealed a significant main effect of condition (*F*(1,111) = 5.364, *p* = 0.022, η_p_^2^ = 0.046), but no main effect of topic (*F*(1,111) = 0.273, *p* = 0.602) or interaction between topic and condition (*F*(1,111) = 0.228, *p* = 0.634). The mean mind attribution score for the pro-god condition was 2.966, for the con-god condition 3.362, for the pro-baseball condition 2.750, and for the con-baseball condition 3.352.

None of our variables were significantly associated with dissonance at baseline, or after viewing and attributing agency toward the robot. However, cognitive dissonance at the time of the belief activation prompt was influenced by participant’s religiosity (Beta = 0.117, 95% *CI*[0.024, 210], *z* = 2.462,* p* = 0.014), offering evidence that more religious individuals experience greater cognitive dissonance in response to the belief prompt. A post hoc investigation of this association identified a significant correlation between religiosity and cognitive dissonance at the time of the prompt, for participants who received the belief activation prompt about God (*r* = 0.314, *p* = 0.015), but not those prompted about baseball (*r* = 0.129, *p* = 0.342). This suggests therefore that the God belief activation exercise induced greater cognitive dissonance in those participants who were more religious.

As in Experiment 1, cognitive dissonance was positively related to attributions of mind, but only dissonance experienced after the prompt (Beta = 0.242, 95% *CI*[0.055,0.563], *z* = 2.381, *p* = 0.017) and robot video (Beta = 0.224, 95% *CI*[0.024,0.401], *z* = 2.206, *p* = 0.027), and not during baseline (Beta = − 0.046, 95% *CI*[− 0.216, 124], *z* = − 0.044, *p* = 0.604). None of the indirect effects were significant, although the indirect Religiosity—> Dissonance Prompt—> Mind Attributions effect was nearly significant (Beta = 0.028, 95% *CI*[− 0.004, 0.061], z = 1.711, *p* = 0.087).

As in Experiment 1, pruning non-significant paths from the mediation model did not alter the significance of any remaining effects.

Because the mind attribution questions used in Experiment 2 yielded a two-factor solution, we ran two additional path analyses, one with a dependent measure reflecting the mean score on the first factor items and one reflecting the mean score on the second factor items. Collectively, the items in the first factor mostly referred to *specific skills* (e.g., recognizing objects, making plans, making decisions). Although some of these items are similar to Gray, Gray and Wegner’s Agency dimension we refer to this subset of questions as specific skills because they also include specific perceptual identification skills and specific cognitive skills such as learning from mistakes and making decisions. The second factor is more related to *broad capabilities* in addition to experiences, so we refer to it as broad skills or characterizations of thinking.

**Fig. 3:**
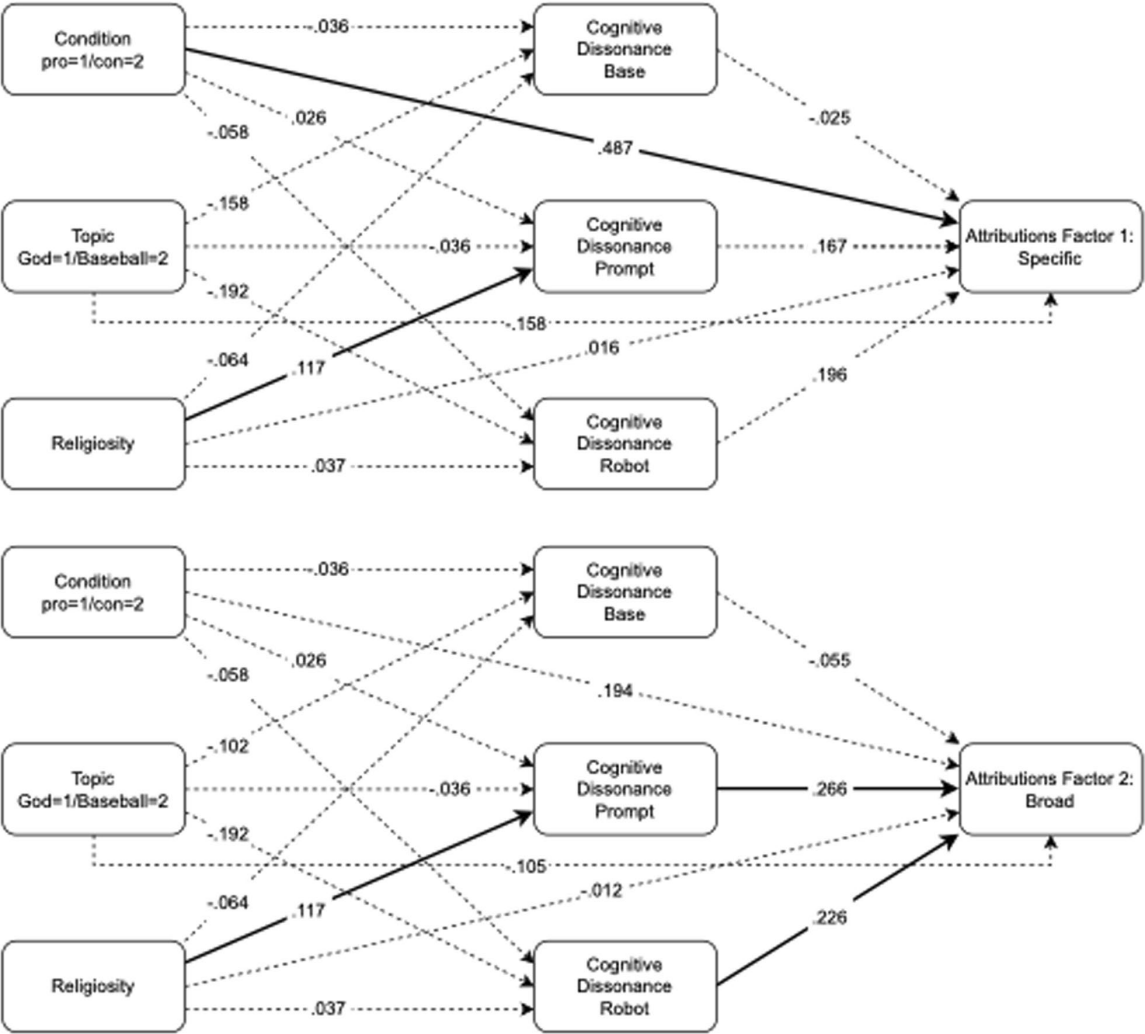
Path models for two mind attribution factors in Experiment 2. Bold lines display significant effects and dashed lines represent non-significant effects. Model coefficients are standardized beta estimates

As shown in Fig. 3, the pattern of results is overall similar for the two measures, but there were some differences. First, the cognitive dissonance measures were numerically stronger predictors for the broad mind attribution factors. The link from cognitive dissonance at the prompt to broad mind attributions was significant (Beta = 0.266, 95% *CI*[0.064, 0.468], *z* = 2.576, *p* = 0.010) while the link from prompt dissonance to the specific attribution was not (Beta = 0.167, 95% *CI*[− 0.037,0.372], *z* = 1.607, *p* = 0.108). Similarly, the link from cognitive dissonance to the robot to broad mind attributions was significant (Beta = 0.226, 95% *CI*[0.023,0.428], *z* = 2.186, *p* = 0.029) while the link to specific mind attributions was not (Beta = 0.196, 95% CI[− 0.008,401], *z* = 1.884, *p* = 0.060). Conversely, the link from condition to the specific attribution factor was significant (Beta = 0.487, 95% *CI*[0.155, 819], *z* = 2.875, *p* = 0.004) while the link to the broad factor was not (Beta = 0.194, 95% *CI*[− 0.135,0.522], *z* = 1.155, *p* = 0.248).

Finally, because the confirmatory factor analyses for the cognitive dissonance questions were inconsistent, we assessed the degree to which the individual questions in the scale predicted mind attributions in a set of correlations with the other significant variables in each regression partialled out. We did this both for Experiment 1 and the significant applications of the questions (to the prompt and to the robot) in Experiment 2. Table 1 shows these results, and it demonstrates first that four of the six questions on the scale produced mostly significant or almost significant correlations with mind attributions, and that none of the questions produced clearly contrasting results. The two questions that seem to have produced the weakest results were the reverse-scored questions, “I was always certain about my responses” and “I never had difficulty putting together all of the facts in this experiment.”

**Table 1:** Correlations between individual items in the cognitive dissonance scale and mind attributions with other significant predictors partialled out

Item	Exp 1	Exp 2	Exp 2
		Prompt	Robot
Always Certain About Resp	.098	.070	.136
Would Change Resp	.145	.230*	.082
Worried Resp Inconsistent	.319***	.146	.247
No Difficulty Putting Together	.105	− .066	.051
Resp Inconsistent With Beliefs	.283**	.218*	.191*
Uncomfortable With Resp	.191*	.157#	.176#

^#^ *p* < .10; *p < .05; **p < .01; ***p < .001

## Discussion

Experiment 2 replicated and extended the findings in Experiment 1. Cognitive dissonance again predicted increased mind attributions, but only when dissonance arose during the experiment and not when reports of dissonance referred to baseline cognitive conflict during the previous week. In addition, Experiment 2 provided evidence that our belief activation exercise did have some impact on cognitive dissonance. Here, participant religiosity predicted greater cognitive dissonance in response to the prompt, and this association was only present for participants who received the God prompt and not the baseball prompt. Finally, we again found a link between prompt-driven arguments and agency attributions, but this time giving reasons someone might *not* believe in God or baseball increased mind attributions in contrast with Experiment 1 where con arguments about God decreased agency attributions. It is interesting to note that the specific agency attributions were more strongly predicted by con arguments but not cognitive dissonance, while the broader attributions showed the reverse pattern because they were more strongly predicted by dissonance and less strongly by the con arguments.

Because the link between the stance (pro/con) participants took and mind attribution was the opposite in Experiment 2 relative to Experiment 1, we further explored the effect. One important difference between the experiments was that we added items to the mind attribution scale in Experiment 2. We therefore assessed the items in Experiment 2 that repeated the items Experiment 1. The mean mind attribution on these items (which assessed the broader attributions) was 2.26 for the pro stance and 2.52 for the con stance, and this difference was not significant (*p* = 0.257, *d* = 0.216). The mean mind attribution on the new items (assessing the specific attributions) was stronger and significant: 3.05 and in the pro condition and 3.61 in the con condition (*p* = 0.011, *d* = 0.484). So, the broad items from Experiment 1 captured decreased agency attribution in the con condition, and no significant difference in Experiment 2, whereas only the items unique to Experiment 2 (i.e., specific) significantly demonstrated the reversed effect. This is roughly consistent with the two-factor analysis of the mind items presented above because four of the five items in Experiment 1 loaded on the second broad factor and this factor captured no significant link between condition and mind attributions.

It is also possible that participants’ interpretation of the broad attribution items changed somewhat when presented in the context of the more specific items. For example, the presence of the specific items, which were more numerous (*n* = 12) than the broad items (*n* = 8), may have induced more specific interpretations of all of the cognitive characteristics. This might support the hypothesis that con arguments lessen broad attributions to agents but increase specific attributions. However, we present this hypothesis as very tentative because it is ad hoc.

More generally, the baseball vs God condition contrast had little effect aside from some differential relationships between participant religiosity and dissonance across the conditions. This might therefore be seen as a failure to confirm the hypothesis that activating beliefs and experiences about topics such as divine agency would increase attributions of mind. However, it remains possible that the manipulation was simply not strong enough to have the expected impact, so future research may consider means of strengthening this manipulation. For example, some forms of accountability can increase motivated reasoning and possibly attitude change (for review see Lerner & Tetlock, 1999) so perhaps a future experiment in which participants are informed that their essays will be read by others who might be influenced their argument (Nel et al., 1969; Hoyt et al., 1972) would induce deeper thought and more cognitive conflict, and provide evidence in support of this hypothesis.

## General discussion

These experiments suggest that cognitive dissonance can increase attributions of mind to artificial agents. This pattern of results is therefore consistent with a selective attribution approach to anthropomorphism, where participants are initially hesitant to attribute minds, but do so upon further reflection (Hymel et al., 2011; Jaeger & Levin, 2016; Levin et al., 2008, 2012, p. 201; Levin et al., 2013a, 2013b; Levin et al., 2013a, 2013b). We also observed that increased religiosity predicted dissonance in response to the concept activation prompt, and that this dissonance was in turn predictive of increased attributions of agency, although the mediated path from religiosity via dissonance to attributions of mind was nonsignificant. This link from religiosity to prompt dissonance was significant only for the God prompt condition. We note that this finding, if confirmed in a setting where sufficient power is available to observe a mediated path from religiosity to agency attribution, may have the potential to explain the mix of findings relating religiosity to agency attribution where some studies reveal this link while others do not. For example, van Elk (2013) observed that agency detection was related to beliefs in the paranormal, but not to traditional beliefs, whereas other researchers (such as Wlodarski & Pearce, 2016) did observe the latter association.

Here, we suggest that cognitive dissonance impacts reasoning about mindedness in several different ways. First, dissonance in response to experiences with a specific attributional target (a robot in this case) predicts attributions of mind. Previously, dissonance arising from expectations about a specific robot’s performance (e.g., tasks where the robot performs especially well or poorly; Levin et al., 2013a, 2013b) impacted mind attributions to that robot. More general research on cognitive dissonance typically also elicits dissonance in response to a specific target, which is often a change in a particular belief or behavior (e.g., Pearce & Cooper, 2021; Stone et al., 1994). However, in this work, we also uncover a broader link between cognitive dissonance and our agency attributions outcome, in which cognitive conflict in response to a prompt to consider a topic other than the target agent nonetheless has accompanied increased attributions of agency. We suggest then that cognitive dissonance is a more general state, which may be influenced by task-specific beliefs as well as conflict around broader related topics. This is consistent with some theories of dissonance such as Simon et al.’s (1995) model of trivialization that reminding participants of other important values can also reduce dissonance (see Harmon-Jones & Harmon-Jones, 2007 for review). However, we note that cognitive dissonance elicited by the prompts in the current study remained relatively low and using the same scale was higher when generated from situation-specific beliefs about a robot that participants interacted with directly (Levin et al., 2013a, 2013b).

In the present work, there are several potential reasons why cognitive dissonance may arise. First, the availability of deep or broad concepts may induce dissonance. Access to broad knowledge of agency concepts (here, regarding religious beliefs) may induce transitions which lead to reconceptualization of existing ideas (Jaeger & Levin, 2016). However, within the problem-solving literature, deep knowledge required to solve problems is typically only used when made accessible in the study context (Chi & VanLehn, 2012). Research from Spatola and Urbanska (2020) suggests an overlap between individuals’ representations of divine and artificial agents, suggesting that available knowledge around religious concepts may transfer to agency representations in non-human agents. In Experiment 2, we found that participant’s religious beliefs predicted cognitive dissonance arising after the belief prompt, but do not show other reliable manipulations of cognitive dissonance. This may suggest that simple availability of deep concepts, while necessary, is not sufficient by themselves to evoke cognitive dissonance. Instead, activation and working-through of these topics may be required, for example by requiring people to compare topics to existing knowledge. Further, the relationship between anthropomorphism and cognitive dissonance may not be clear-cut and unidirectional. For example, Kim and Ryoo (2022) show that anthropomorphized chatbots are more effective at inducing dissonance and changing subsequent COVID-preventative behaviors. As such, there is evidence both that dissonance increases anthropomorphizing, but also that anthropomorphizing mediates the effectiveness of dissonance induction.

Another potentially interesting, but somewhat inconsistent, finding in the present work is that generating arguments against a nominally normative belief increased attributions of agency in Experiment 2. In Experiment 2, we found that this relationship was not specific to the religious belief prompt and also arises from negative arguments about the control prompt (why should baseball not be considered America’s national sport). As such, this may reflect a more general form of conceptualization of alternative ideas or counter-normative reasoning. The transition model of agency cognition (Jaeger & Levin, 2016) argues that reconceptualization of default ideas represents a deeper, elaborative form of interaction that may lead to conceptual change. Through the path modeling in the current work, we suggest that this path may be separate from the conceptual change evoked by cognitive dissonance. Consistent with this distinction, the generalized link from con arguments to attribution in Experiment 2 was stronger for the specific attribution items, while the link from dissonance to attribution was stronger for the broad attribution items.

Finally, we note that our data were collected some time ago, in the spring and summer of 2016, and this might be a concern for a topic subject to rapid technological change. Although it is likely that people’s understanding of machine agency has evolved over time, and that this may be an interesting topic of study, we do not think that these changes have fundamentally altered people’s reasoning about agency during the last several years. First, the basic concepts about agency that underlie our judgments likely stem from forms of social cognition such as theory of mind that underlie reasoning about a wide range of human agents (Hortensius & Cross, 2018). These social constraints are likely broad and have their roots in early development, so it is unlikely that they have undergone a fundamental revolution over the last decade. Second, both the general idea of robots, and specific examples of robots and other intelligent machine agents have been available to most of our US-based participant pool consistently for quite some time and do not seem to have changed markedly since 2016. If one searches google trends for “robot” as a search term, results demonstrate a consistent relative number of searches from 2004 until 2023 with only a small increase during this time. Also, specific household robots such as Roomba were developed in 2002 and were commonly searched for with a spike during the Christmas season just before our data collection. The level of interest since then has increased somewhat, but it peaked in 2018 and decreased since then. Interest in self-driving cars also increased during the two years previous to our data collection, then peaked at a slightly higher level, and declined slightly thereafter. Thus, we believe that our research explores concepts about technology that may be evolving, but not radically changing as people integrate new information with existing knowledge developed and refined over the last several decades of relatively consistent interest in these topics. That being said, investigating the impact of socio-cognitive processes with increasingly prevalent agents in the form of chatbots and AI prompts will surely be of interest for future study.

Cognitive dissonance is one of many factors involved in attribution of human qualities to non-human agents. When evaluating interactions with robots, we may make a holistic range of socio-cognitive judgements beyond mind attribution: for example, trustworthiness (Bigman & Gray, 2018; Waytz et al., 2014), confidence (Dietvorst et al., 2014), morality (Swiderska & Kuester, 2020), and satisfaction with the interaction (Xie et al., 2022). Recent findings suggest convergence between neural regions active when anthropomorphizing artificial agents and regions involved when attributing trustworthiness, socialness, and beliefs toward agents (see Hortensius & Cross, 2018 for a review). The interaction of cognitive dissonance with these wider socio-cognitive processes in human–robot interaction is still underexplored, and we suggest future work explore these relationships.

## Summary and conclusions

In these experiments, we demonstrate that mind attributions can be influenced by situation-specific cognitive dissonance. Uncertainty toward a robotic agent promotes increased attribution of human-like qualities to that agent. Curiously, evoking feelings of uncertainty outside of the immediate context of the robotic agent still increases attributions of agency. These findings may inform the contexts in which humans interact with autonomous systems for a wide array of emerging technology.

## Data Availability

The datasets generated and analyzed during the current study are available from the corresponding author upon reasonable request.

## Appendix—additional analyses and full factor analysis results.

EFA’s for religiosity and agency questions as these were not previously used measures, and one-factor CFA’s for the cognitive dissonance questions as we have used those before. For the religiosity scales in both Experiments 1 and 2, the EFA’s produce one-factor solutions:

## Experiment 1: religiosity

**Table:**

Factor Loadings		
	Factor 1	Uniqueness
ImportFaith	0.948	0.101
PrayAdj	0.934	0.128
BeliefGod	0.815	0.336
ConfidenceTrad	0.703	0.506
AttendAdj	0.682	0.535

Applied rotation method is promax

**Table:**

Factor Characteristics							
	Unrotated solution				Rotated solution		
Eigenvalues SumSq. Loadings Proportion var.Cumulative SumSq. Loadings Proportion var. Cumulative							
Factor1	3.676	3.395	0.679	0.679	3.395	0.679	0.679

## Experiment 2: religiosity

**Table:**

	Factor 1	Uniqueness
FaithImport	0.943	0.112
FaithGuide	0.938	0.120
Pray_FIX	0.903	0.185
BelieveInGod	0.887	0.212
FaithHist	0.646	0.583
AttendFIX	0.618	0.617

Applied rotation method is promax.

**Table:**

Factor characteristics							
	Unrotated solution				Rotated solution		
Eigenvalues SumSq. Loadings Proportion var. Cumulative SumSq. Loadings Proportion var. Cumulative							
Factor1	4.419	4.171	0.695	0.695	4.171	0.695	0.695

In Experiment 1, our agency measure produced a one-factor solution:

**Table:**

Factor Loadings		
	Factor 1	Uniqueness
BotMind	0.868	0.246
BotIntent	0.771	0.406
BotConsc	0.759	0.424
INV_BotEmo	0.612	0.626
INV_BotWill	0.609	0.629

Applied rotation method is promax.

**Table:**

Factor characteristics							
	Unrotated solution				Rotated solution		
Eigenvalues SumSq. Loadings Proportion var. Cumulative SumSq. Loadings Proportion var. Cumulative							
Factor1	3.104	2.669	0.534	0.534	2.669	0.534	0.534

In Experiment 2, our measure of the expanded agency produced a two-factor solution. The two-factor solution in the expanded experiment 2 measure is listed below. Inspection of the items in the two factors suggest that one factor (factor 2) represents relatively broad characterizations of the robots’ experience. This factor includes ratings of the degree to which the robot might have consciousness, a mind, emotions, free fill, and beliefs. The other factor seems to reflect more specific attributions of skills to the robot. This factor includes ratings of the degree to which the robot makes decisions, plans, recognizes things, and learns.

**Table:**

Factor Loadings			
	Factor 1	Factor 2	Uniqueness
Recognizes	0.907		0.348
Perceives	0.889		0.365
Strategies	0.885		0.380
Navigates	0.827		0.486
Solves	0.781		0.371
Decisions	0.714		0.442
Plans	0.649		0.414
Learns	0.646		0.487
ConsidersAlt	0.634		0.502
Knows	0.601		0.418
Understands	0.459		0.648
Intentions	0.448		0.452
Emotions		1.014	0.197
FreeWill		1.006	0.156
Beliefs		0.988	0.230
Frustrated		0.868	0.298
Consciousness		0.786	0.349
Abstract		0.749	0.367
Mind		0.683	0.389
Preferences		0.522	0.448
Communicates			0.714

Applied rotation method is promax.

**Table:**

Factor Characteristics							
	Unrotated solution				Rotated solution		
Eigenvalues SumSq. Loadings Proportion var. Cumulative SumSq. Loadings Proportion var. Cumulative							
Factor1	10.487	10.097	0.481	0.481	6.443	0.307	0.307
Factor2	2.765	2.441	0.116	0.597	6.095	0.290	0.597

We note that although these items grouped with different factors, the correlation between subscores derived from the means of these items (and not including the “communicates” item that did not group with either factor) was + 0.62 suggesting that it is reasonable to combine them in a full measure. We have therefore retained the same primary outcome measure in Experiment 2. However, below, we present two path models, one with a score derived from each measure. This analysis does show some differences, but only in degree.

We have also included one-factor CFA’s for our measures of cognitive dissonance. The fits for the scale in experiment 1 are mediocre:

## Model fit

Chi-square test.

**Table:**

Model	X^2^	df	P
Baseline model	149.926	15	
Factor model	32.868	9	< 0.001

The estimator is ML.

## Additional fit measures

Fit indices.

**Table:**

Index	Value
Comparative Fit Index (CFI)	0.823
Tucker–Lewis Index (TLI)	0.705
Bentler–Bonett Non-normed Fit Index (NNFI)	0.705
Bentler–Bonett Normed Fit Index (NFI)	0.781
Parsimony Normed Fit Index (PNFI)	0.468
Bollen’s Relative Fit Index < RFI)	0.635
Bollen’s Incremental Fit Index (IFI)	0.831
Relative Noncentrality Index (RNI)	0.823

The fits for the three applications of the scale in Experiment 2 are a bit better but still not particularly good;

Cognitive Dissonance base:

## Additional fit measures

Fit indices

**Table:**

Index	Value
Comparative Fit Index (CFI)	0.929
Tucker–Lewis Index (TLI)	0.881
Bentler–Bonett Non-normed Fit Index (NNFI)	0.881
Bentler–Bonett Normed Fit Index (NFI)	0.903
Parsimony Normed Fit Index (PNFI)	0.542
Bollen’s Relative Fit Index (RFI)	0.838
Bollen’s Incremental Fit Index (IFI)	0.930
Relative Noncentrality Index (RNI)	0.929

Cognitive Dissonance prompt:

## Additional fit measures

**Table:**

Fit indices	
Index	Value
Comparative Fit Index (CFI)	0.819
Tucker–Lewis Index (TLI)	0.699
Bentler–Bonett Non-normed Fit Index (NNFI)	0.699
Bentler–Bonett Normed Fit Index (NFI)	0.796
Parsimony Normed Fit Index (PNFI)	0.478
Bollen’s Relative Fit Index (RFI)	0.661
Bollen’s Incremental Fit Index (IFI)	0.824
Relative Noncentrality Index (RNI)	0.819

Cognitive Dissonance robot:

## Additional fit measures

**Table:**

Fit indices	
Index	Value
Comparative Fit Index (CFI)	0.895
Tucker–Lewis Index (TLI)	0.825
Bentler–Bonett Non-normed Fit Index (NNFI)	0.825
Bentler–Bonett Normed Fit Index (NFI)	0.874
Parsimony Normed Fit Index (PNFI)	0.525
Bollen’s Relative Fit Index (RFI)	0.791
Bollen’s Incremental Fit Index (IFI)	0.897
Relative Noncentrality Index (RNI)	0.895

These fits led us to test the performance of the individual items on the scale in our models in place of the entire scale. These are depicted in the tables below:

Correlations between individual items in the cognitive dissonance scale and attributed agency with other significant predictors partialled out. Note that items 1 and 4 are reverse scored such that high ratings indicate high levels of cognitive dissonance.

**Table:**

Item	Exp 1	Exp 2	Exp 2
		Prompt	Robot
1. Always Certain About Resp	0.098	0.070	0.136
2. Would Change Resp	0.145	0.230*	0.082
3. Worried Resp Inconsistent	0.319***	0.146	0.247**
4. No Difficulty Putting Together	0.105	− 0.066	0.051
5. Resp Inconsistent With Beliefs	0.283**	0.218*	0.191*
6. Uncomfortable With Resp	0.191*	0.157#	0.176#

# *p* < 0.10; **p* < 0.05; ***p* < 0.01;****p* < 0.001.

Correlations between individual items in the cognitive dissonance scale and religiosity:

**Table:**

Item	Exp 2
	Prompt
1. Always Certain About Resp	0.168#
2. Would Change Resp	0.115
3. Worried Resp Inconsistent	0.115
4. No Difficulty Putting Together	0.183*
5. Resp Inconsistent With Beliefs	0.162#
6. Uncomfortable With Resp	0.117

# *p* < 0.10; **p* = 0.05; ***p* < 0.01;****p* < 0.001.

The overall pattern of correlations lead us to two conclusions. First, although the cognitive dissonance questions may not be a particularly strong single factor, the individual items in the scale are not so heterogeneous as to be hiding contrasting effects – almost all of the items in almost all of the experiments produce small correlations in the same direction. Second, the reverse-scored items, (1 and 4 above) seem to be less effective predictors of agency attributions than the others. Also, the parameter estimates for factor loadings on these two items are the lowest for cognitive dissonance in all three cases (in the tables below, they are labeled as the PromptFacts and the PromptCertain items and they are not reverse scored):

## Parameter estimates

**Table:**

Factor loadings								
							95% Confidence Interval	
Factor	Indicator	Estimate	Std. Error	z-value	P		Lower	Upper
Factor 1	PromptChange	0.991	0.125	7.927	< 0.001		0.746	1.236
	Promptlnconsist	1.162	0.122	9.521	< 0.001		0.923	1.401
	PromptBeliefs	1.095	0.123	8.888	< 0.001		0.854	1.337
	PromptUncomfortable	1.239	0.147	8.436	** < **0.001		0.951	1.527
	PromptFacts	− 0.621	0.148	− 4.208	< 0.001		− 0.910	− 0.332
	PromptCertain	− 0.682	0.142	− 4.817	< 0.001		− 0.960	− 0.405

### Parameter estimates

**Table:**

Factor loadings								
							*95%* Confidence Interval	
Factor	Indicator	Estimate	Std. Error	Z–value	P		Lower	Upper
Factor 1	BaseChange	1.256	0.134		< 0.001		0.993	1.519
	Baselnconsist	1.278	0.145	S.845	< 0.001		0.995	1.561
	Base Facts	− 0.6SS	0.157	− 4.385	** < **0.001		− 0.995	− 0.3 SO
	BaseCertain	− 0.914	0.141	− 6.493	< 0.001		− 1.190	− 0,638
	Base Beliefs	LI 70	0.119	9.819	< 0.001		0.936	1,403
	Ba seU n co mf o rtable	1.297	0.127	10.242	< 0.001		1.049	1,546

#### Parameter estimates

**Table:**

Factor loadings							
						9596 Confidence Interval	
Factor	Indicator	Estimate	Std. Error	z-value	P	Lower	Upper
Factor 1	Ro botch an ge	1.248	0.J21	10.351	< 0.001	1.012	1.485
	Robotlnconsist	1.165	0.141	8.265	< 0.001	0.889	1.442
	Robot Beliefs	1.251	0.110	11.335	< 0.001	1.035	1.468
	Ro botU n co mf o rtable	1.327	0.129	10.254	< 0.001	1.073	1.580
	RobotCertain	− 0.B4S	0.140	− 6.081	< 0.001	− 1.122	− 0.575
	Robot Facts	− 0.939	0.158	− 5.949	< 0.001	− 1.248	− O.630

Given these patterns, we created a new version of the cognitive dissonance variable with only the four positively-scaled items and reran the CFA’s. In these cases, the fit indices are better. Base Dissonance: CFIT: 0.974, RMSEA = 0.054, X2 = 7.831, Prompt dissonance: CFIT = 0.964, RMSEA = 0.066, X2 = 8.651, Robot dissonance: CFIT:1.00, RMSEA = 0.00, X2 = 1.132.

Given this, we reran the main model for experiment 2 with the 4-item versions of the cognitive dissonance questionnaire and observed very similar results:

Comparing this figure to the one in the paper with the full version reveals only very small differences in path coefficients, and in all cases but one, significant results stay significant and non-significant results stay nonsignificant. The one exception is that the link from religiosity to cognitive dissonance at the prompt changes from Beta = 0.117, *p* = 0.014 to Beta = 0.082, *p* = 0.086.
